# Melting Gold

**Published:** 1883-07

**Authors:** 


Melting Gold.—In melting coarse gold, blow the fire to a great heat and stir the metal with a stick of carbon, or the long stem of a tobacco pipe, to prevent honey-combing. If steel or iron filings get into gold while melting, throw in a piece of saltpeter the size of a walnut; it will attract the iron or steel from the gold into the flux; or, sublimate of mercury will destroy the iron or steel. To cause gold to roll well, melt with a good heat, add a tablespoonful of sal ammoniac and charcoal, equal quantities, both pulverized, stir up well, put on the cover for two minutes, and pour.—*The Jewelers' Journal.*

